# National outbreak of Shiga toxin-producing *Escherichia coli* O145:H28 associated with pre-packed sandwiches, United Kingdom, May–June 2024

**DOI:** 10.1017/S0950268824001729

**Published:** 2024-12-27

**Authors:** Orlagh Quinn, Yanshi, Grace King, Ann Hoban, Clare Sawyer, Amy Douglas, Anaïs Painset, Andre Charlett, Andrew Nelson, Carys Rees, Chloe Byers, Christopher Williams, Colin Brown, Kitty Mohan, Claire Brown, Claire Jenkins, Claire Neill, Genna Leckenby, Lesley Larkin, Lesley Allison, Oluwakemi Olufon, Sema Nickbakhsh, Trish Mannes, Thomas Inns, Sooria Balasegaram

**Affiliations:** 1 UK Health Security Agency, London, UK; 2 Public Health Wales, Cardiff, UK; 3 Public Health Agency, Belfast, Northern Ireland; 4 Public Health Scotland, Glasgow, UK; 5 Scottish Escherichia coli O157/STEC Reference Laboratory, Edinburgh, UK

**Keywords:** Shiga toxin-producing *Escherichia coli*, outbreaks, United Kingdom, pre-packaged sandwiches, epidemiology, surveillance

## Abstract

Shiga toxin-producing *Escherichia coli* (STEC) is a group of bacteria that causes gastrointestinal illness and occasionally causes large foodborne outbreaks. It represents a major public health concern due to its ability to cause severe illness which can sometimes be fatal. This study was undertaken as part of a rapid investigation into a national foodborne outbreak of STEC O145. On 22 May 2024, United Kingdom (UK) public health agencies and laboratories identified an increase in stool specimens submissions and patients testing positive for Shiga toxin-producing *E. coli* (STEC). Whole genome sequencing (WGS) identified serotype O145:H28 *stx2a/eae* belonging to the same five single nucleotide polymorphism (SNP) single linkage cluster as the causative agent. By 3 July 2024, 288 cases had been linked to the cluster. Most cases were adults (87%) and females (57%), 49% were hospitalized with a further 10% attending emergency care. Descriptive epidemiology and analytical studies were conducted which identified consumption of nationally distributed pre-packed sandwiches as a common food exposure. The implicated food business operators voluntarily recalled ready-to-eat sandwiches and wraps containing lettuce on 14 June 2024.

## Background

Enhanced surveillance systems for STEC across the UK combine detailed clinical and epidemiological data (including symptoms, travel, food, and animal exposure) collected on enhanced surveillance questionnaires (ESQ) with the microbiological characterization of strains using whole genome sequencing (WGS) [1]. Diagnostic laboratories report presumptive cases of STEC based on PCR or culture, directly to health protection teams (and to local authorities in Wales), who undertake public health management including collection of information via the STEC ESQ within 48 h.

Faecal specimens from suspected cases of STEC and/or isolates of STEC are referred to the UKHSA Gastrointestinal Bacteria Reference Unit (GBRU) in London or the Scottish *E. coli* Reference Laboratory (SERL) in Edinburgh. Faecal specimens testing positive for STEC by PCR are cultured and all isolates of STEC are sequenced. Characterization includes clonal complex and sequence typing, serotyping, *stx* typing, and SNP typing [2].

## Descriptive epidemiology

**Figure 1.**
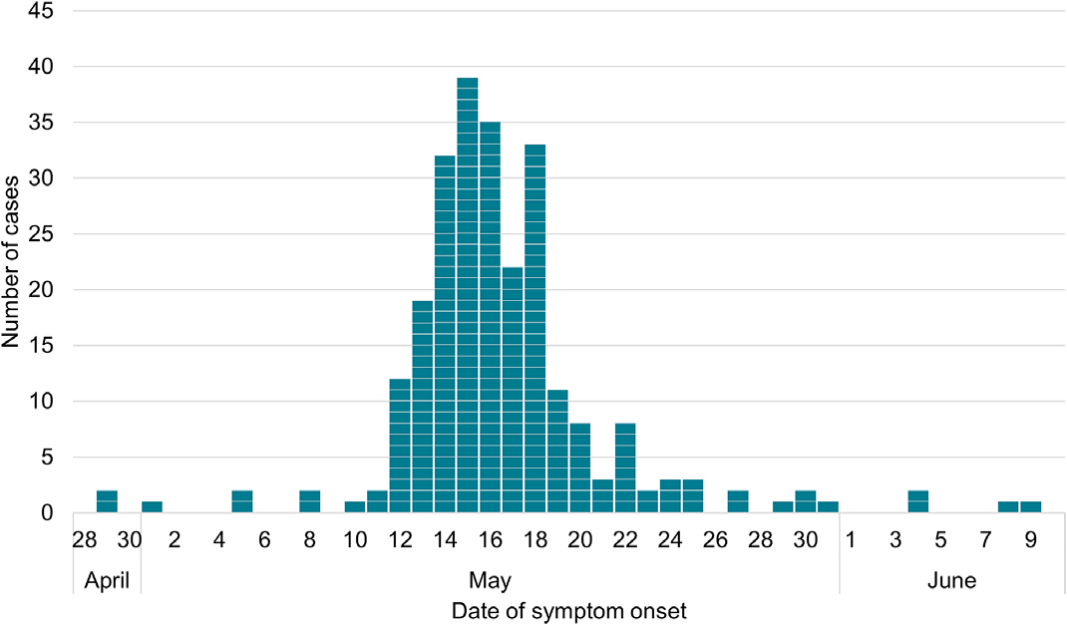
Confirmed primary cases by date of symptom onset (*n* = 248). *Note*: Onset date is unavailable for 38 cases.

**Figure 2.**
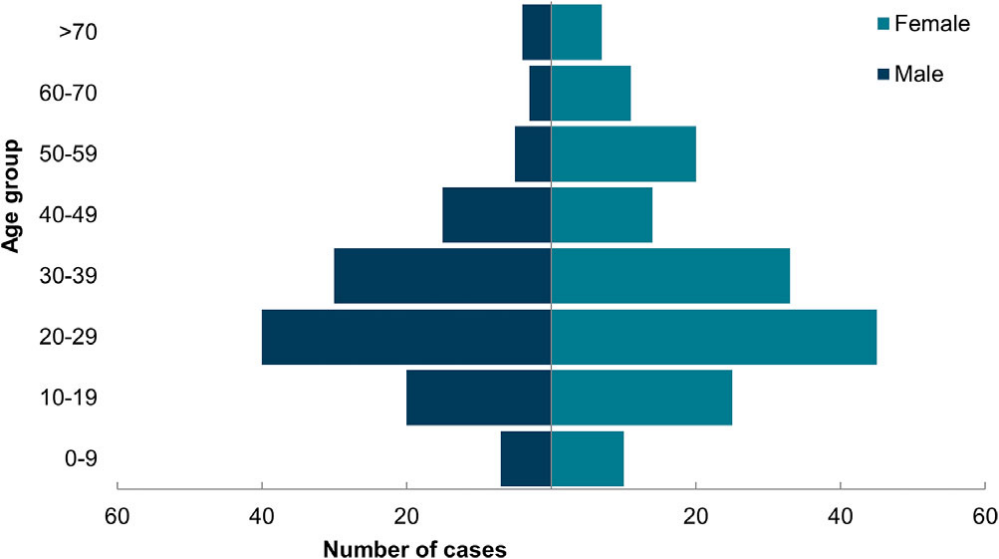
Age/sex pyramid of cases (*n* = 288).

Of the 288 reported cases, confirmed to be linked by WGS within a five SNP cluster, 286 were symptomatic primary cases; four (two in England and two in Scotland) were symptomatic secondary cases. Symptom onset dates of the primary cases ranged from 29 April 2024 to 17 June 2024 (Figure 1). Primary cases had a median age of 29 years (range: 1–89) and were predominantly female (57%) (Figure 2). Cases were geographically dispersed across the UK. For cases where information was available (*n* = 263), 49% of cases were hospitalized, and 80% of symptomatic cases reported bloody diarrhoea. There were nine cases of haemolytic uraemic syndrome (HUS), and two deaths among these confirmed cases (Table 1).

**Table 1.** Clinical information including reported symptoms (*n* = 263)

Clinical information	No. cases	%
Diarrhoea	253	96%
Blood in stool	210	80%
Nausea	147	56%
Vomiting	100	38%
Abdominal pain	238	90%
Fever	79	30%
Other symptoms	44	17%
Attended A&E	103	39%
Admitted to hospital	129	49%
Haemolytic uraemic syndrome	9	3%
Death	2	1%

*Note*: Data is unavailable for 25 cases.

## Hypothesis generation

Data collected through UKHSA’s National Enhanced STEC Surveillance System (NESSS), and complementary enhanced surveillance in Scotland, Wales, and Northern Ireland, were reviewed. A case–case study was conducted using English cases in the outbreak cluster as cases, and other English STEC cases as controls. Cases and controls with the same age profile and with sample dates from April 2024 to May 2024 were included. The analysis indicated pre-packaged sandwiches as a possible hypothesis, OR 4.91 (95%CI 1.51–15.1, *P* 0.004). A similar study in Wales identified the same hypothesis. In logistic regression with subsequent cases (outbreak cases *n* = 59, control cases *n* = 64) the final model included pre-packaged sandwiches (OR 3.88, 95%CI 1.65–9.57, *P* 0.002), iceberg lettuce (OR 2.99, 95%CI 1.24–7.48, *P* 0.016), and eating out (OR 2.17, 95%CI 0.91–5.37, *P* 0.08) as significant exposures for the outbreak cases.

In total, 11 of 15 cases interviewed with a trawling questionnaire reported consuming pre-packaged sandwiches from different national retailers. Additionally, given the incubation period of STEC, the epi curve indicated that the exposure period for the cases must have been very brief and therefore suggestive of a short shelf-life product. Based on the generated hypothesis, we undertook an analytical study using targeted questionnaires with more detail on pre-packaged sandwiches, eating out, and salad consumption. Case data were correct as of 09 June 2024 (*n* = 43).

## Analytical studies

Following the initial case–case studies, outbreak cases were compared to two sources of control. All controls were frequency matched to cases in age bands and reported no travel outside of the UK in the week before data collection. Controls in study 1 were cases of *Salmonella* residents in the UK with a notification date from 01 April 2024 to 09 June 2024, (*n* = 63) and were asked about their food histories for the week prior to their onset with *Salmonella.* Controls in study 2 were recruited by a Market Panel [3] and reported no diarrhoea in the previous week (06 June 2024).

In both analytical studies, variables significantly associated with outbreak case status (odds ratio (OR) > 1 and *P* < 0.1) in single variable analysis and age and sex as *a priori* potential confounding variables were included in a multivariable Firth logistic regression model using a forward step approach for model construction with all models including the potential confounders.

In the multivariable models, cases were significantly more likely to have consumed pre-packaged sandwiches containing lettuce (for Study 1 Model 1 OR 7.1, 95%CI 2.3–21.5, *P* 0.001; and for Study 2 Model 1 OR 4.8, 95%CI 1.9–12.0, *P* < 0.001) (Table 2).

**Table 2.** Multivariable analysis of estimated odds ratios of infection with STEC O145 t5.206

Study 1 Model 1: STEC O145 t5.206 cases with Salmonella cases as case-controls.							
Exposure	Cases (*n* = 43)		Controls (*n* = 63)		aOR*	95% CI**	*P* value
	*n*	%	*n*	%			
Age (ref: 11–18 year olds)							
Age: 19–29 year olds	25	58	18	29	4.4	0.9–21.3	0.06
Age: 30–70 year olds	12	28	33	52	2.3	0.5–10.8	0.3
Sex (ref: female)							
Male	15	35	22	35	1.2	0.4–3.1	0.3
Consumed a pre-packaged sandwich containing any lettuce leaf	25	58	6	10	7.1	2.3–21.5	0.001
Consumed a pre-packaged sandwich containing BLT/bacon	14	33	1	2	6.7	1.0–43.4	0.05

Study 2 Model 1: STEC O145 t5.206 cases with market panel controls							
Exposure	Cases (*n* = 43)		Controls (*n* = 93)		aOR*	95% CI**	*P* value
	*n*	%	*n*	%			
Age (ref: 11–18 year olds)							
Age: 19–34 year olds	25	58	43	46	2.1	0.5–8.4	0.3
Age: 35–70 year olds	12	28	37	40	1.1	0.3–4.5	0.9
Sex (ref: female)							
Male	15	35	35	38	1.0	0.4–2.6	>0.9
Consumed a pre-packaged sandwich containing BLT/bacon	14	33	3	3	6.4	1.7–24.7	0.007
Consumed a pre-packaged sandwich containing any lettuce leaf	25	58	15	16	4.8	1.9–12.0	0.001
Mince beef	15	35	18	19	2.7	1.0–6.9	0.04

Study 2 Model 2: Assessing lettuce in sandwich variable using market panel controls							
Exposure	Cases (*n* = 43)		Controls (*n* = 93)		aOR*	95% CI**	*P* value
	*n*	%	*n*	%			
Age (ref: 11–18 year olds)							
Age: 19–34 year olds	25	58	43	46	1.8	0.5–6.2	0.7
Age: 35–70 year olds	12	28	37	40	1.0	0.3–3.5	>0.9
Sex (ref: female)							
Male	15	35	35	38	1.2	0.5–2.9	0.7
Sandwich containing lettuce (ref: no sandwich eaten)							
Consumed sandwich containing lettuce	25	58	15	16	7.1	3.0–17.5	<0.001
Consumed sandwich without lettuce	3	7	13	14	1.0	0.3–3.9	>0.9
Consumed sandwich possibly containing lettuce	3	7	10	11	1.5	0.4–6.0	0.5

Study 2 Model 3: Assessing lettuce whilst dining out using market panel controls							
Exposure	Cases (*n* = 43)		Controls (*n* = 93)		aOR*	95% CI**	*P* value
	*n*	%	*n*	%			
Age (ref: 11–18 year olds)							
Age: 19–34 year olds	25	58	43	46	1.8	0.5–6.1	0.3
Age: 35–70 year olds	12	28	37	40	1.0	0.3–3.5	>0.9
Sex (ref: female)							
Male	15	35	35	38	1.2	0.5–2.8	0.7
Lettuce when dining out (ref: no sandwich eaten with lettuce or no dining out meal including lettuce)							
Consumed sandwich containing lettuce	25	58	15	16	7.2	3.1–16.8	<0.001
Consumed lettuce as part of a meal out (but not in a sandwich)	3	7	10	11	1.4	0.4–5.4	0.6

* aOR: Adjusted odds ratio.

** CI: confidence interval.

In Study 2 Model 2 (Table 2), cases were significantly more likely to have consumed a prepackaged sandwich with lettuce compared to any other type of sandwich or no sandwiches (OR 7.1, 95%CI 3.0–17.5, *P* < 0.001).

A separate model to compare lettuce consumed in sandwiches to lettuce consumed when eating out showed the latter was not significant either (Study 2 Model 3) (Table 2).

## Food chain and environmental investigation

Food chain investigations identified the sandwich producer that supplied the retailers during May 2024. The sandwich producer had sourced lettuce from farms in England. Further food chain investigations are ongoing.

## Control measures

On 13 June 2024, supplier/producer A and B voluntarily recalled various sandwiches, wraps, and salads because of possible contamination with *E. coli.* Consumers who had bought the products listed were advised not to eat them, and to return them to the store where they were purchased for a full refund. A further recall occurred on 15 June 2024 by supplier/producer C.

Epipulse communications to ECDC indicated of the 13 European countries who replied, none were affected.

## Discussion

Since 2015, across the UK notifications of STEC O157:H7 have declined and STEC O26:H11 and STEC O145:H28 have emerged as a significant cause of gastrointestinal infectious disease and HUS [1, 4, 5]. Since 2020, STEC O145:H28 has consistently been in the top five most common STEC serotypes reported in England and Scotland [5, 6].

Outbreaks of STEC infection have previously been associated with pre-packed sandwiches and salad vegetables, mainly lettuce, in the UK and elsewhere [7–10]. Ready-to-eat salad vegetables are vulnerable to contamination with pathogens at the pre-harvest level via flooding, rainwater run-off, or irrigation water containing animal faeces [11]. Current methods for washing and decontaminating fresh produce cannot guarantee that pathogens, if present, will be removed. STEC can adhere to leaves and become internalized within leafy vegetables [12]. The application of controls to minimize the risk of faecal contamination during growing, handling, and processing is therefore of fundamental importance in ensuring the safety of fresh produce.

Monitoring PCR results provided an early indication of the outbreak and surveillance data case–case analysis facilitated rapid hypothesis generation. Subsequent analytical studies established a precise definition and exploration of the composite product. Interdisciplinary collaboration and cooperation from the major food retailers led to voluntarily removal of the implicated product from sale thus reducing the risk of an on-going transmission.

Number of confirmed cases decreased since 31 May 2024, but the outbreak has not yet been declared over. Food chain investigations are ongoing, and the location of the animal reservoir and/or mechanisms of crop contamination are currently unclear. Possible routes of contamination include a failure in control measures protecting the crop from agricultural run-off, contamination of water or growing materials used in lettuce production, or contaminated seeds. The implicated lettuce is a UK product, and no cases are known to have occurred outside the UK. Nevertheless, the international community should be aware of this vehicle of infection and monitor for possible ongoing cases linked to this outbreak of STEC O145:H28, as similar routes of contamination may occur in other countries.
